# Neural evidence of deprioritizing to-be-forgotten information in visual working memory

**DOI:** 10.3389/fcogn.2024.1404909

**Published:** 2024-07-30

**Authors:** Katherine C. Moen, Melissa R. Beck, Scarlett Horner, Steven G. Greening

**Affiliations:** ^1^Department of Psychology, University of Nebraska at Kearney, Kearney, NE, United States; ^2^Department of Psychology, Louisiana State University, Baton Rouge, LA, United States; ^3^Department of Psychology, University of Manitoba, Winnipeg, MB, Canada

**Keywords:** visual working memory, directed forgetting, forgetting mechanisms, functional imaging, passive forgetting, active forgetting

## Abstract

**Introduction:**

Although evidence supports the effective use of a cue to forget an encoded stimulus, the mechanisms of this forgetting are not well understood. Evidence from item-method directed forgetting in long-term memory reveals greater prefrontal and parietal activation for information that is cued to be forgotten. Activation in those brain regions is typically associated with increased effort and cognitive control.

**Method:**

To test the mechanism of directed forgetting in visual working memory, we used stimuli that rely on distinct brain regions such as faces and buildings and varied memory stability. Participants completed a directed forgetting task with faces and buildings, and memory stability was manipulated by presenting some stimuli repeatedly throughout the study, and other stimuli were only presented once.

**Results and discussion:**

Functional magnetic resonance imaging (fMRI) results from the parahippocampal place area suggest that to-be-remembered buildings elicit higher activation than to-be-forgotten buildings. In addition, dorsolateral prefrontal cortex activation changed throughout the trial period, possibly suggesting that the cue led to information being dropped from visual working memory, or through a shift in attention, as occurs with the retro-cue paradigm. Several explanations for these results are discussed.

## 1 Introduction

Visual working memory (VWM) is a capacity limited memory system that can be optimized by prioritizing task relevant information (Baddeley, 1992, 2003). One way that information is prioritized is by forgetting encoded information that is no longer task relevant. Therefore, to efficiently maintain task-relevant information in VWM, information that is no longer relevant to the ongoing task may be forgotten or deprioritized (Williams and Woodman, 2012). Although it is well documented that cueing some encoded information as task relevant and other information as task irrelevant can improve memory performance for the cued information, the mechanisms supporting this prioritization are largely unknown. For example, the information that can be forgotten because it is no longer task relevant may be actively forgotten, requiring cognitive resources (Wylie et al., 2008; Nowicka et al., 2011; Rizio and Dennis, 2013), or passively forgotten through the removal of cognitive resources (Maxcey and Woodman, 2014; Zwissler et al., 2015; Souza and Oberauer, 2016; Taylor and Hamm, 2016; Dagry and Barrouillet, 2017; Sasin et al., 2017; Schneegans and Bays, 2018). In addition, information stored within VWM may be forgotten through a different mechanism than information that can rely on a more stable memory store (e.g., long term memory). The goal of the current study was to determine the mechanism (active or passive) of directed forgetting in VWM by manipulating memory stability and using stimuli that rely on distinct brain regions.

How individuals remember or forget information in VWM is often measured with the directed forgetting (DF) paradigm (MacLeod, 1975). In a traditional VWM DF task, participants encode an array of a small number of stimuli (typically two to six) for a short time (e.g., 1,000 ms). For cue trials, the cue (e.g., an arrow pointing to one side of the display) to maintain a subset of stimuli (i.e., to-be-remembered [TBR] stimuli) is presented during a delay (maintenance) interval, and the other stimuli are no longer relevant to the current task (i.e., to-be-forgotten [TBF] stimuli). For no-cue trials, participants attempt to maintain all of the stimuli. After the delay interval, participants are tested (on TBR stimuli for cue trials and all stimuli for no-cue trials) typically by reporting if a change occurred to the stimuli from the encoding display to the test display. Participants typically have higher accuracy on cue trials than no-cue trials (cuing effect; MacLeod, 1975; Williams and Woodman, 2012; Williams et al., 2013; Gunseli et al., 2015; Van Moorselaar et al., 2015), suggesting that the TBF stimuli were forgotten, and that maintaining less information maximizes processing efficiency (MacLeod, 1975; Anderson et al., 1994; Festini and Reuter-Lorenz, 2014).

Many long-term memory (LTM) directed forgetting studies use item-method DF tasks (MacLeod, 1975; Anderson and Green, 2001; Wylie et al., 2008; Nowicka et al., 2011; Rizio and Dennis, 2013; Zwissler et al., 2015; Fawcett et al., 2016). In an item-method DF task, participants study stimuli one at a time and then receive an immediate cue instructing them to either remember or forget the preceding stimulus. After participants encode several stimuli, and sometimes after an additional delay of several minutes (to ensure information is in LTM), participants complete a recognition memory test on all the stimuli. Item-method DF tasks allow for the direct testing of TBF information, which is more challenging with an array-based DF task typically used in VWM research. In array-based DF, you can only ask about the TBF stimuli once, before participants begin to ignore the cue (Moen et al., 2019). In the current study we aimed to overcome testing difficulties in array-based methods to examine if directed forgetting in VWM is active or passive by measuring brain activation for TBR and TBF items that activate distinct brain regions.

Research on forgetting in LTM has supported both active and passive forgetting as mechanisms of DF (Rizio and Dennis, 2013; Anderson and Hanslmayr, 2014; Maxcey and Woodman, 2014; Zwissler et al., 2015; Souza and Oberauer, 2016; Taylor and Hamm, 2016; Dagry and Barrouillet, 2017; Sasin et al., 2017; Schneegans and Bays, 2018). Supporters of both perspectives agree that individuals focus available cognitive resources on TBR information when presented with a cue. However, the two perspectives differ in how TBF information is “forgotten.” The active suppression hypothesis is characterized by actively inhibiting TBF information. Thus, individuals use cognitive resources to both remember TBR information and forget TBF information. Moreover, if suppression is successful, individuals will not be able to remember the TBF information if probed to do so (Rizio and Dennis, 2013; Anderson and Hanslmayr, 2014). Conversely, passive forgetting involves focusing all cognitive resources on TBR information leading to a weaker but existent memory trace for TBF information. If probed to recall TBF information, individuals may report some information, but the memory trace would be weak (Maxcey and Woodman, 2014; Zwissler et al., 2015; Souza and Oberauer, 2016; Taylor and Hamm, 2016; Dagry and Barrouillet, 2017; Sasin et al., 2017; Schneegans and Bays, 2018; Moen et al., 2019). However, as cognitive resources are more limited within VWM compared to LTM (Atkinson and Shiffrin, 1968), forgetting may occur differently in VWM compared to LTM.

Most evidence supporting active forgetting as the mechanism of directed forgetting comes from item-method DF tasks in long-term memory (Fawcett and Taylor, 2008; Wylie et al., 2008; Nowicka et al., 2011; Rizio and Dennis, 2013). Active forgetting is characterized by actively inhibiting TBF information, and this is supported by neurological evidence. There is greater prefrontal (middle frontal gyrus and right superior frontal gyrus) and parietal (precuneus and right inferior parietal lobe) activation following a cue to forget compared to a cue to remember in LTM item-method DF tasks. Activation in those brain regions is typically associated with increased effort and cognitive control (Wylie et al., 2008; Nowicka et al., 2011; Rizio and Dennis, 2013). These neurological findings suggest that directed forgetting in LTM is not a passive process, but rather an active, effortful inhibition of TBF information. However, successful active suppression should lead to failures to remember the TBF information if probed to report it, but this is not what has been found (Nowicka et al., 2011; Rizio and Dennis, 2013). Therefore, the active inhibition process may not be fully successful in removing information from memory, making it hard to distinguish from a passive process when looking at behavioral evidence alone.

Passive forgetting is characterized by focusing more cognitive resources on the TBR information than the TBF information, leading to weaker, but still existent, memory traces for TBF information. Thus, if probed to recall TBF information, individuals may be able to report some information, but the memory trace for the TBF information would be weaker than for the TBR information (Maxcey and Woodman, 2014; Zwissler et al., 2015; Souza and Oberauer, 2016; Taylor and Hamm, 2016; Dagry and Barrouillet, 2017; Sasin et al., 2017; Schneegans and Bays, 2018). Zwissler et al. (2015) conducted an experiment using an item-method DF task but compared performance on “remember” and “forget” cue trials to trials when participants received no cue to remember or forget (neutral stimuli). They argued that based on the active forgetting hypothesis, TBF stimuli would be remembered less accurately than or equivalent to neutral stimuli, because of the purposeful forgetting process. They found the traditional DF effect in that TBR stimuli were remembered with higher accuracy than TBF stimuli, but TBF stimuli were remembered with higher accuracy than neutral stimuli. Zwissler et al. (2015) argued that their results suggest that participants were selectively rehearsing only the TBR stimuli, and therefore experienced passive forgetting for the TBF stimuli. Because participants were not actively suppressing nor rehearsing the TBF stimuli, they still had some memory for that information, but not at the level of TBR stimuli (Zwissler et al., 2015). Similarly, other research found that memory for TBF stimuli is stable and above chance, suggesting that TBF information is not purposefully inhibited or removed from memory, but instead is less accessible (Dagry and Barrouillet, 2017).

It is unknown if the active forgetting process found for LTM using item-method DF tasks will be found for array DF tasks used to test forgetting in VWM. There may be different types of forgetting due to the different types of tasks used. Encoding and maintenance in VWM are active processes (LaRocque et al., 2014). For example, maintaining items and item location-binding processes necessary in an array-based task may require active processing (van Lamsweerde et al., 2015; Hitch et al., 2020; Brady and Störmer, 2022). It is possible that the active process of encoding and maintaining information in VWM leads to passive forgetting in VWM. If active processes are required to maintain information in VWM, the removal of active processing could be sufficient for forgetting and no active suppression would be needed.

Another key body of literature related to the process of passive forgetting is the shift in attention that occurs by using retro-cues. In retro-cue tasks participants are presented with stimuli (simultaneous or sequentially) and then the location of one stimulus is cued, after the encoding window, as being the location of the to-be-tested stimulus. The validity of the retro-cue varies, depending on the study, but it is often over 75% valid, to encourage participants to use the cue. Valid retro-cues typically lead to higher accuracy and faster response times than invalid retro-cues or no-cue trials. One important aspect of the retro-cue effect is the shifting of attention to the cued stimulus. This shifting of attention is thought to improve memory for the cued stimulus (Griffin and Nobre, 2003; Janczyk and Berryhill, 2014), although this attention shift can be effortful and require practice (Zerr et al., 2021). The retro-cue literature supports the idea of passive forgetting, but the mechanism of that “forgetting” is specifically a shift in attention to the cued stimulus. We revisit this possibility and the similarity between retro-cues and directed forgetting in the discussion (see Section 4.2).

Neuroimaging may help distinguish between the possible mechanisms of directed forgetting in VWM (active vs. passive forgetting), by using stimuli that rely on distinct brain regions such as faces and buildings (Beck et al., 2001; Gazzaley et al., 2005; Schmitz et al., 2010; Detre et al., 2013; Cohen and Tong, 2015). The fusiform face area (FFA) is in the lateral temporal lobe along the ventral stream of the visual pathway, and consistently shows greater activation in response to faces than other stimuli such as buildings or objects (Kanwisher et al., 1997). The parahippocampal place area (PPA) is located near the hippocampus in the posterior infero-medial temporal lobe and is medial to the FFA and shows greater activation in response to naturalistic scenes and buildings (Epstein et al., 1999). Several studies have utilized these areas to investigate a wide variety of research questions and have revealed that while the FFA and PPA are located in close proximity, they are distinct, separable brain regions (Epstein et al., 1999; Beck et al., 2001; Gazzaley et al., 2005; Schmitz et al., 2010; Detre et al., 2013; Cohen and Tong, 2015). Further, it has been shown that directing attention to faces activates the FFA, while directing attention to places activates the PPA (Lepsien and Nobre, 2007).

Using neurologically distinct stimuli allowed us to examine the neural mechanisms for TBR and TBF information on the same trial. For example, if participants are presented with one face and one building, and are cued to remember the building, the face is now TBF. Regardless of the hypothesized forgetting mechanism, participants shift cognitive resources to the TBR stimulus following the cue. Thus, there may be greater activation in the brain region associated with the TBR stimulus than the TBF stimulus on cue trials compared to no-cue trials. However, the predictions for each hypothesis differ depending on the memory stability (i.e., prior existence of a memory template in LTM) and dorsolateral prefrontal cortex activation (DLPFC). Active suppression will likely result in increased DLPFC activation after the cue, due to the increased cognitive effort associated with purposeful forgetting (Wylie et al., 2008; Nowicka et al., 2011; Rizio and Dennis, 2013). In contrast, passive forgetting will most likely manifest with less activation in the DLPFC, due to a lower VWM load following the cue (Thompson and Taylor, 2015).

To further examine how more stable memory representations impact forgetting, the current study also manipulated whether the TBF information was new information (presented for the first time during the experiment) or old information (previously presented in a different portion of the experiment) to test how stable memories are forgotten (TBF status). Old TBF stimuli may result in greater activation overall during the DF task due to increased familiarity (Weibert and Andrews, 2015; Henson, 2016). Representations of the previously presented stimuli are more likely to be supported by LTM, allowing a test of the forgetting mechanisms in VWM vs. LTM. Maintaining information in VWM requires cognitive resources (van Lamsweerde et al., 2015). However, cognitive resources can be freed up from maintaining VWM representations if they can be offloaded to LTM (Bartsch and Shepherdson, 2022). Prioritization of VWM resources may not require suppression of representations that can be supported by LTM. Therefore, the mechanism of forgetting could vary for information more easily offloaded to LTM (previously encountered information).

The present study combined a VWM forgetting task with fMRI to adjudicate between the two prominent theories of forgetting by testing the following hypotheses. First, regardless of the mechanism of DF, we predicted greater activation in the perceptual region associated with the cued stimulus compared to the region associated with the non-cued stimulus, because participants shift cognitive resources to the TBR stimulus following the cue. Second, if forgetting in VWM is an active process we will observe increased DLPFC activation after the cue due to purposeful forgetting (Wylie et al., 2008; Nowicka et al., 2011; Rizio and Dennis, 2013). However, if forgetting in VWM is a passive process we will observe less activation in the DLPFC following the cue due to a lower VWM load (Thompson and Taylor, 2015). Third, previously viewed stimuli may result in more stable VWM representations due to increased familiarity (Weibert and Andrews, 2015; Henson, 2016), and may be forgotten differently than novel stimuli.

## 2 Method

### 2.1 Design

We employed a 2 × 2 repeated measures design, manipulating cue presence (cue, no-cue) and the TBF relevance (old vs. new TBF stimulus).

### 2.2 Participants

Twenty participants completed the current study. Sample size was based on the effect sizes from previous work (Moen et al., 2019). G^*^Power was used to calculate the required sample size, by using Cohen's *d* from the cueing effect (no-cue vs. cue) for change trials (*d* = 0.67). Based on the power analysis, 20 participants were required to achieve an estimated power of 0.80. The effect sizes used to estimate sample size were taken from behavioral data. However, other neuroimaging studies examining change detection with similar stimuli have utilized much smaller sample sizes (e.g., Beck et al., 2001 had 10 participants). Participants were recruited from undergraduate psychology courses at Louisiana State University and received partial course credit for participation.

### 2.3 Materials

Two hundred fifty Caucasian, female faces and 250 buildings were adapted from various image databases (Princeton University Library, 2022). All stimuli were presented in gray scale. Following the procedure of Cohen and Tong (2015), there was a 250-pixel circle encompassing each stimulus centered on the nose (for faces) or on the center of the building to make the stimuli as similar as possible and reduce extraneous details (e.g., clothing) from the face stimuli. Eyebrows and hairlines were visible on the faces to equate perceptual complexity with the buildings. The visual angles of all stimuli were 6.8 × 7.2 degrees (width × height).

### 2.4 Procedure

Prior to entering the scanner, participants reviewed and signed the consent form, and were given instructions regarding the scanning process and each task. Participants practiced the DF task outside of the scanner. Once participants were in the scanner, an anatomical scan (5 min) was followed by the localizer task (4 min) and the DF task (35 min).

#### 2.4.1 Localizer task

Participants were presented with 36 female faces and 36 places (houses and buildings) individually for 1,500 ms, followed by a 1,500 ms fixation cross. Participants were told to remember the images for a memory test later on, outside of the scanner.^1^ The stimuli were presented in eight blocks (nine stimuli each). Each block only contained one type of stimulus (faces or buildings), but the order of blocks was randomized for each participant. The localizer task served two purposes: first, it was used to create regions of interest for the FFA and PPA, and a subset of stimuli presented during the localizer task served as the “old” TBF stimuli in the DF task.

#### 2.4.2 Directed forgetting task

**Figure 1:**
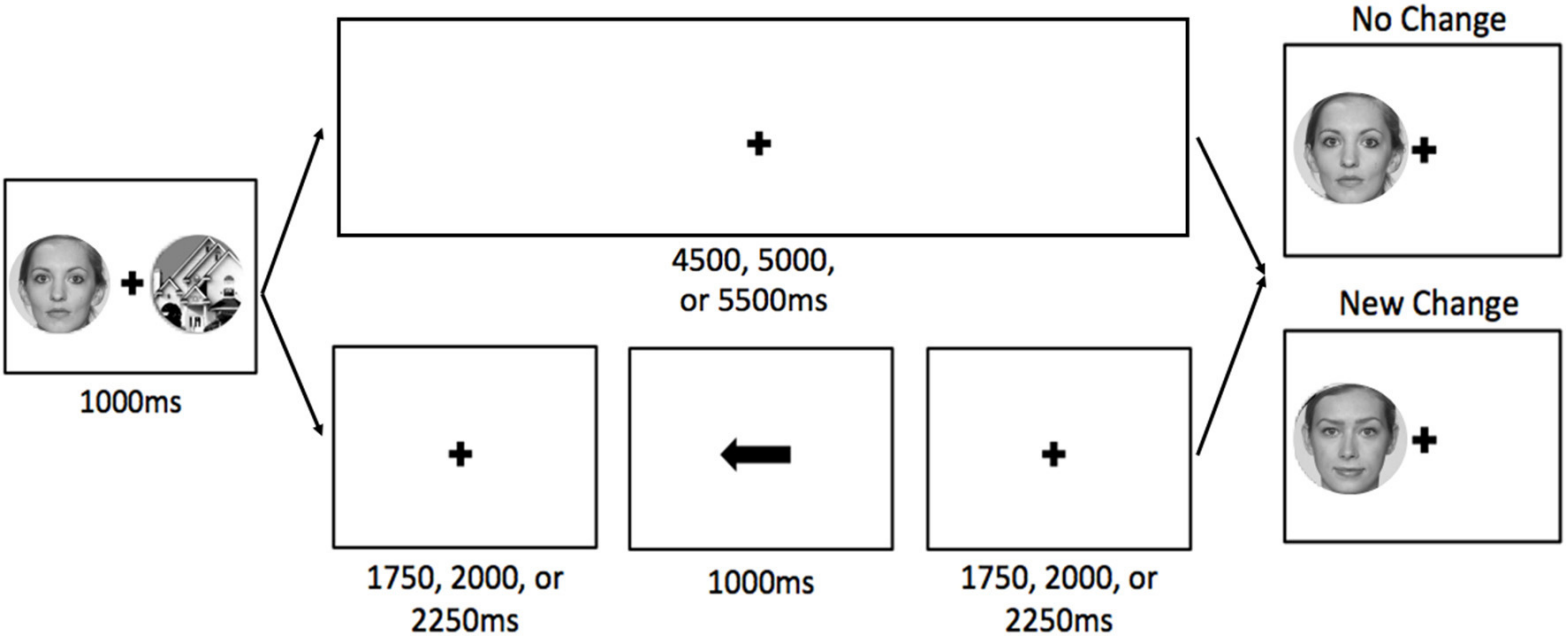
Visual depiction of a typical trial sequence for no-cue **(top)** and cue **(bottom)** trials. For cue trials, the cue was always valid. The stimuli are enlarged to show detail. The faces in this example are from the Face Research Lab London Set (DeBruine, Lisa; Jones, Benedict (2017). Face Research Lab London Set. Dataset. https://doi.org/10.6084/m9.figshare.5047666.v5).

Each trial began with a 4,000 ms fixation point, during which participants were instructed to relax and wait for the next trial. These inter-trial intervals were used for baseline activation. Participants were then presented with a face on one side of the display and a building on the other side for 1,000 ms (Figure 1). On no-cue trials, both stimuli were always new (not presented during the localizer task). On cue trials, the TBF stimulus was either new (50%) or old (50%; presented during the localizer task) and the TBR stimulus was always new. For cue trials (75% of trials), a fixation cross was presented for 1,750, 2,000, or 2,250 ms (to avoid predictability and jitter timing for the analyses), followed by an arrow pointing to the left or right side of the display, indicating the side of the display that would be tested, and thus, should be remembered. The cue was always valid, and pointed to the side of the display that would be tested. The arrow remained on the display for 1,000 ms. Following the cue, a fixation cross remained on the display for 1,750, 2,000, or 2,250 ms before the post-change display appeared, which contained only one stimulus. For no-cue trials (25% of trials), the fixation cross after the pre-change display remained on the display for 4,750, 5,000, or 5,250 ms. Regardless of cue presence, when the post-change display appeared, participants responded whether a change occurred with a button box. The post-change display always contained one stimulus, and it was either identical to the pre-change display (i.e., no-change, half of trials) or a new stimulus belonging to the same stimulus group (e.g., face presented on left during pre-change, a different face was used post-change). See Figure 1 for an example of the trial sequence. Participants completed a total of six runs, each containing 24 trials (six no cue trials and 18 cue trials [half old TBR stimuli, half new TBR stimuli]). Each run lasted approximately 5.5 min.

#### 2.4.3 Long-term memory recognition test

After participants completed the directed forgetting task, they exited the scanner and completed a long-term memory task. They were shown stimuli from the localizer and directed forgetting tasks and were asked to indicate if they saw each stimulus once, twice, or not at all during the tasks in the scanner. Participants were presented with 126 pictures (half faces, half places) in a random order. Half of the stimuli were TBR stimuli and half were TBF stimuli.

### 2.5 Imaging parameters

FMRI data were collected on a GE 3-T Magnet with a 32 channel MR Instruments head coil at Pennington Biomedical Research Center. The structural image was acquired using a three-dimensional magnetization-prepared rapid acquisition gradient (MPRAGE) sequence (TR = 9.252 ms, TE = 3.788 ms, flip angle = 8°, 224 × 256 matrix, phase encoding direction right to left). Functional scans contained 36 slices with a voxel resolution of 3.5 × 3.5 and a slice thickness of 3 mm. Functional scans were acquired using a Gradient Echo EPI, echo-planar imaging sequence with the following parameters: repetition time (TR) = 2,000 ms; echo time (TE) = 25 ms; flip angle, 90°. The frequency field of view was 22.4 and phase field of view was 1.0. Each scan began with three dummy volumes to account for equilibrium effects, and those dummy volumes were discarded from the analyses during preprocessing. The specific number of volumes varied for each portion of the experiment with the localizer task containing 130 volumes and each run of the directed forgetting task containing 160 volumes.

#### 2.5.1 fMRI preprocessing and whole-brain univariate analysis

FMRI data processing was carried out using FEAT (FMRI Expert Analysis Tool) Version 6.00, part of FSL (FMRIB's Software Library, www.fmrib.ox.ac.uk/fsl). Registration of the functional images to both the high-resolution (T1-weighted) structural image and the standard space image was carried out using FLIRT (Jenkinson and Smith, 2001; Jenkinson et al., 2002). The following pre-statistics processing was applied: motion correction using MCFLIRT (Jenkinson et al., 2002); slice-timing correction using Fourier-space time-series phase-shifting; non-brain removal using BET (Smith, 2002); spatial smoothing using a Gaussian kernel of FWHM 5 mm; high-pass temporal filtering (Gaussian-weighted least-squares straight line fitting, with sigma = 50.0 s). Following preprocessing, we ran three distinct first-level models using the General Linear Model (GLM) at the single-subject level. Notably, all three GLMs included several nuisance regressors including six standard motion correction parameters along with 18 extended motion parameters, and motion censoring regressors for any volume with >0.9 mm framewise displacement (Siegel et al., 2014) using the fsl_motion_outliers function. Lastly, a whole brain analysis was conducted on the localizer task data to examine brain activation in areas that were more active when viewing faces than buildings, and more active for buildings than faces. To isolate the PPA and FFA, we conducted a group-level analysis using FLAME stage 1 (Beckmann et al., 2003). The resulting *Z* (Gaussianised *T/F*) statistic images were thresholded using clusters determined by *Z* > 3.1 and a (corrected) cluster significance threshold of *p* = 0.05 (Worsley, 2001). Group-level masks were then created for each region-of-interest (ROI), one for the left PPA, one for the right PPA, and one for the right FFA to examine brain activation during the DF task using a ROI approach.

#### 2.5.2 GLM1

The first GLM (GLM1) was designed to separate cue and no-cue trials. Cue trials were further separated into the stimulus that was cued (cued face, cued building) and the status of the TBF stimulus (old, new). We used a double-gamma hemodynamic response function (HRF) to model each of the conditions of interest. For cue trials, the HRF was modeled to include period from the onset of the pre-change display to the offset of the final fixation cross, immediately before the post-change array onset (i.e., the response screen). For no-cue trials, we examined from the onset of the pre-change display to the offset of the fixation cross, immediately before the post-change array onset. This was done to equate the total time frame examined for cue and no-cue trials. As nuisance regressors, we also modeled the post-change array (i.e., the response phase) for each condition. This ensured that our estimation of the blood-oxygenation-level-dependent (BOLD) change attributable to our conditions of interest was not confounded by BOLD activity from responding. A second-level analysis was performed to average each experimental run during the DF task for each participant. This was completed using a fixed effects model, by forcing the random effects variance to zero in FLAME (FMRIB's Local Analysis of Mixed Effects; Beckmann et al., 2003). Following the analysis of the functional localizer data (see below), activation was analyzed separately for the FFA, left PPA, and right PPA with a 2 × 2 repeated measures ANOVA with cued stimulus (face cued, building cued) and TBF status (old, new) as the factors.

#### 2.5.3 GLM2

The second GLM (GLM2) was designed to delineate between pre-cue vs. post-cue activity on cued trials. The part of the trial that was pre-cue included the onset of the pre-change display to the fixation preceding the arrow cue. The part of the trial that was post-cue included the cue arrow and the fixation immediately following. This factor was combined with the factors of stimulus cued (cued face, cued building) and the status of the TBF stimulus (old, new), which resulted in a total of eight conditions of interest. We also included regressors of no interest for no-cue trials and the response phase of all trials. A second-level analysis was performed to average each experimental run during the DF task for each participant. This was completed using a fixed effects model, by forcing the random effects variance to zero in FLAME (FMRIB's Local Analysis of Mixed Effects; Beckmann et al., 2003). For GLM2, we also ran a whole-brain group-level analyses using FLAME (FMRIB's Local Analysis of Mixed Effects) stage 1 (Beckmann et al., 2003). The resulting *Z* (Gaussianised *T/F*) statistic images were thresholded using clusters determined by *Z* > 2.3 and a (corrected) cluster significance threshold of *p* = 0.05 (Worsley, 2001). The primary purpose of this analysis was to identify BOLD differences from pre-cue to post-cue irrespective of the cued-stimulus and the status of the TBF stimulus.

#### 2.5.4 FIR analysis

To explore potential differences between cue and no-cue trials and to better understand the time course of brain activation that resulted from the group-level analysis of GLM2, we conducted a third first-level GLM using Finite Impulse Responses (FIR) rather than convolving our response model to the HRF, similar to previous research (Lehmann et al., 2006; Schmitz et al., 2010; Grégoire et al., 2023). The FIR analyses allowed us to determine where in the trial period the BOLD signal changed in the DLPFC. We modeled the whole trial window from stimulus onset to beyond the post-change display into the intertrial interval. Specifically, we include 7 bins (i.e., FIRs) per trial, each lasting 2 seconds (i.e., the duration of a TR). A second-level analysis was performed to average each experimental run during the DF task for each participant. This was completed using a fixed effects model, by forcing the random effects variance to zero in FLAME (FMRIB's Local Analysis of Mixed Effects; Beckmann et al., 2003). For the FIR analysis, we predicted that activation would differ for cue and post cue trials throughout the time course of the trial.

## 3 Results

### 3.1 Behavioral results

**Figure 2:**
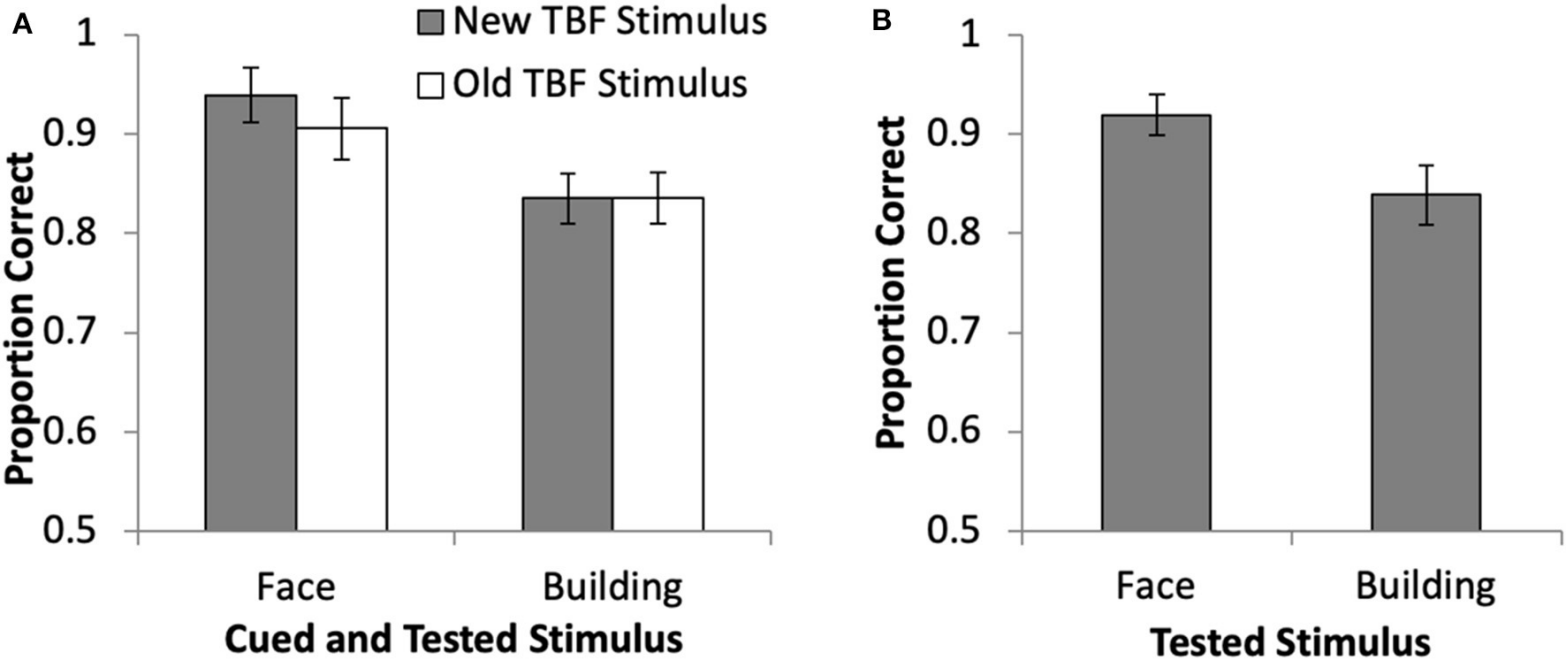
Behavioral results from the directed forgetting task in the current study for cue trials **(A)** and no-cue trials **(B)**. Error bars represent 95% confidence intervals.

Accuracy during the DF task was examined with a 2 × 2 repeated measures ANOVA to compare the cued stimulus (face, building) and TBF status (old, new) for cue trials (Figure 2A). Results revealed a significant main effect of stimulus type (face, building), *F*_(1, 19)_ = 34.13, *p* < 0.01, $\eta_{\text{p}}^{2}$ = 0.64, in that accuracy was significantly higher when the face was cued (*M* = 92.12%, *SD* = 11.77%) than when the building was cued (*M* = 83.64%, *SD* = 11.87%). There was no main effect of TBF status, *F*_(1, 19)_ = 2.07, *p* = 0.17, $\eta_{\text{p}}^{2}$ = 0.10, and no interaction, *F*_(1, 19)_ = 3.20, *p* = 0.09, $\eta_{\text{p}}^{2}$ = 0.14. The proportion correct for no-cue trials when a face or building was tested (Figure 2B) was compared with a paired samples *t*-test, and revealed higher accuracy when the face was tested than when the building was tested, *t*_(19)_ = 3.31, *p* = 0.004. Using paired samples *t*-tests, cue trials were compared to no-cue trials for each stimulus type and TBF status separately and revealed no differences between no-cue trials and any of the cue trial types (*p*s > 0.26). The lack of a difference between cue and no-cue trials does not replicate previous DF research (Williams and Woodman, 2012; Williams et al., 2013; Moen et al., 2019). Overall, these results suggest that faces were remembered more accurately than buildings, and the cue did not increase accuracy. However, it is possible that accuracy was not a sensitive enough measure to detect differences among the various trial types. Thus, neuroimaging data were essential to determine if brain activation changed because of stimulus type and TBF status.

**Figure 3:**
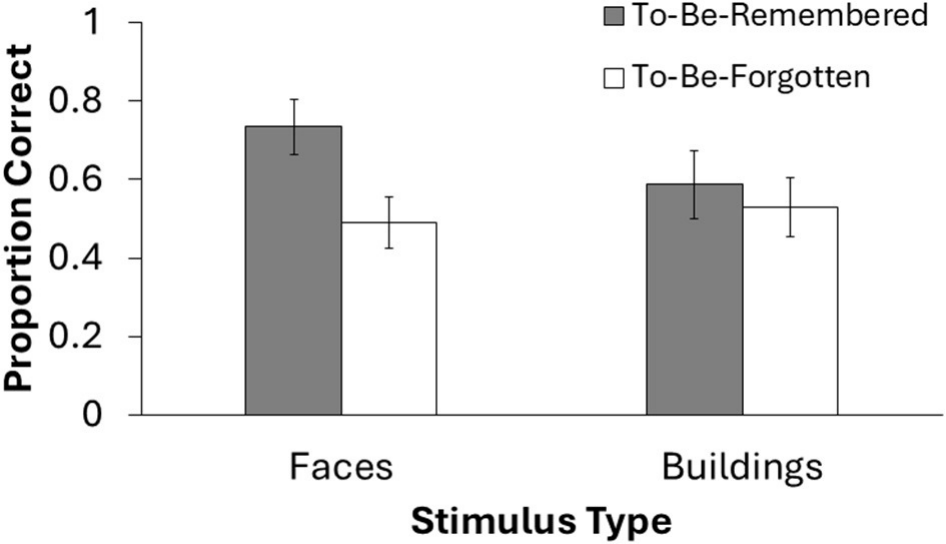
Behavioral results from the long-term memory task at the end of the experimental session. Only 15 participants completed this test due to time restraints. Error bars represent 95% confidence intervals.

Finally, we analyzed data from the long-term memory task that took place outside of the scanner, at the end of each session. Fifteen participants completed this post-test. Five participants did not complete the post-test due to time constraints. To analyze these data, we conducted a 2 × 2 Repeated Measures ANOVA on memory accuracy to compare stimulus type (face, place) and DF status (TBR, TBF). Results revealed no significant main effect of stimulus type, *F*_(1, 14)_ = 1.01, *p* = 0.33, $\eta_{\text{p}}^{2}$ = 0.07. However, there was a significant main effect of DF status, *F*_(1, 14)_ = 32.56, *p* < 0.001, $\eta_{\text{p}}^{2}$ = 0.70, suggesting that participants were more likely to remember TBR stimuli compared TBF stimuli. Additionally, there was a significant interaction between DF status and stimulus type, *F*_(1, 14)_ = 20.22, *p* < 0.001, $\eta_{\text{p}}^{2}$ = 0.59. This interaction was driven by higher accuracy for TBR faces (*M* = 0.73, *SD* = 0.14) compared to TBF faces (*M* = 0.49, *SD* = 0.17), *t*_(14)_ = 6.53, *p* < 0.001, whereas the difference between TBR places (*M* = 0.59, *SD* = 0.13) and TBF places (*M* = 0.53, *SD* = 0.15) was trending toward significance, *t*_(14)_ = 1.94, *p* = 0.075. See Figure 3 for a visual depiction of these data. Overall, these results suggest that the TBR stimuli were ultimately more likely to be retrieved from LTM than the TBF stimuli, especially for face stimuli.

### 3.2 Neuroimaging results

#### 3.2.1 Left PPA activation

**Figure 4:**
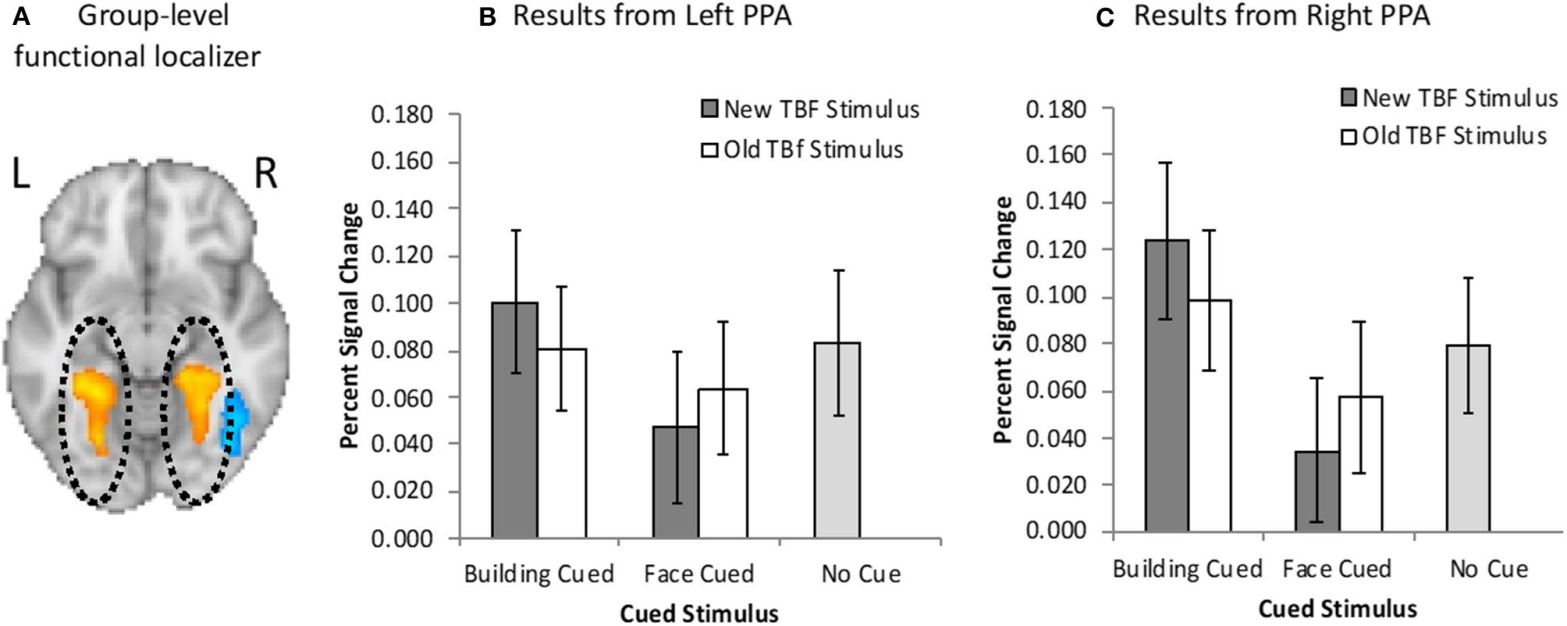
**(A)** Bilateral PPA activation (orange/yellow) resulting from the group-level functional localizer task. Neuroimaging results from the left **(B)** and right **(C)** PPA when buildings were cued or faces were cued, and the TBF stimulus was new or old. Activation on no-cue trials is added for comparison. Error bars represent 95% confidence intervals.

For GLM1, we conducted a 2 × 2 repeated measures ANOVA with cued stimulus (face cued, building cued) and TBF status (old, new) as the factors. Results revealed a main effect of the cued stimulus, *F*_(1, 19)_ = 5.76, *p* = 0.03, $\eta_{\text{p}}^{2}$ = 0.23, no main effect of TBF status, *F*_(1, 19)_ = 0.03, *p* = 0.87, $\eta_{\text{p}}^{2}$ = 0.001, but there was a significant interaction between the cued stimulus and TBF status, *F*_(1, 19)_ = 6.01, *p* = 0.03, $\eta_{\text{p}}^{2}$ = 0.024 (Figure 4B). We conducted paired samples *t*-tests to examine the significant interaction. The interaction was driven by greater left PPA activation on trials when buildings were cued than when faces were cued, but only when the TBF stimulus was new, *t*_(19)_ = 3.63, *p* = 0.002. There were no differences between the type of stimuli cued when the TBF stimulus was old, *t*_(19)_ = 0.96, *p* = 0.35. These results suggest that when all the information presented on a given trial is new, participants are able to prioritize the cued information, and deprioritize the non-cued information, leading to a change in PPA activation.

We further examined Left PPA activation by comparing activation on no-cue trials to cue trials. When the face was cued, activation was significantly higher than no-cue trials, but only when the TBF stimulus was new, *t*_(19)_ = 2.53, *p* = 0.021. This suggests that participants were less likely to maintain a new building when the face was cued than no-cue trials. However, when the building was cued, activation did not differ from no-cue trials, regardless of if the corresponding TBF stimulus was old, *t*_(19)_ = 1.08, *p* = 0.30 or new, *t*_(19)_ = 0.11, *p* = 0.91. There was also no difference in activation between old TBF buildings and new buildings on no-cue trials, *t*_(19)_ = 1.22, *p* = 0.24. Full results are available in Figure 4A.

For GLM2, we conducted a 2 × 2 × 2 repeated measures ANOVA with cued stimulus (face cued, building cued), TBF status (old, new), and time (pre-cue, post-cue). Results revealed a significant main effect of time, *F*_(1, 19)_ = 28.42, *p* < 0.001, $\eta_{\text{p}}^{2}$ = 0.60, suggesting greater Left PPA activation before the cue compared to after the cue. There was a significant three-way interaction among the tested variables, *F*_(1, 19)_ = 4.71, *p* = 0.04, $\eta_{\text{p}}^{2}$ = 0.20. There were no other significant main effects and no significant interactions (*p*s > 0.13). We conducted several paired-sample *t*-tests to determine the source of the three-way interaction. There was a significantly higher Left PPA activation after the cue for new buildings compared to new faces, *t*_(19)_ = 2.47, *p* = 0.02. These results are not surprising, as participants should prioritize the cued stimulus, which would result in increased activation in the PPA when buildings were cued compared to when faces were cued. Additionally, we hypothesized that it would be easier for participants to prioritize the cued stimulus when the TBF stimulus was new. No other *t*-tests were statistically significant (*p*s > 0.11).

Overall, these results revealed greater left PPA activation when buildings were cued, than when faces were cued, but only when the TBF stimulus was new. Participants were less able to effectively utilize the cue to prioritize the cued information when the TBF stimulus was old. These results suggest that more stable memory representations (old TBF stimuli) were less effectively deprioritized compared to less stable memory representations (new TBF stimuli). This pattern of results may be due to old stimuli being maintained by LTM rather than VWM.

#### 3.2.2 Right PPA activation

For GLM1, results revealed a main effect of the cued stimulus, *F*_(1, 19)_ = 20.16, *p* < 0.01, $\eta_{\text{p}}^{2}$ = 0.52, and no main effect of TBF status, *F*_(1, 19)_ = 0.03, *p* = 0.88, $\eta_{\text{p}}^{2}$ = 0.001, but there was a significant interaction between the cued stimulus and TBF status, *F*_(1, 19)_ = 6.27, *p* = 0.02, $\eta_{\text{p}}^{2}$ = 0.025 (Figure 4C). We conducted paired samples *t*-tests to examine the significant interaction. The interaction was driven by greater right PPA activation on trials when buildings were cued than trials when faces were cued, regardless of whether the TBF stimulus was new, *t*_(19)_ = 5.16, *p* < 0.001, or old, *t*_(19)_ = 2.37, *p* = 0.03. However, the difference in PPA activation for cued faces and cued buildings was significantly larger on trials when the TBF stimulus was new, *t*_(19)_ = 2.50, *p* = 0.022.

Finally, we compared right PPA activation on cue trials to no-cue trials, and found greater activation for when buildings were cued, *t*_(19)_ = 3.20, *p* = 0.005, than no-cue trials, and lower activation when faces were cued than no-cue trials, *t*_(19)_ = 3.02, *p* = 0.007, but only for trials when the TBF stimulus was new. There were no differences in activation between no-cue trials and cued buildings, *t*_(19)_ = 1.18, *p* = 0.25, or cued faces, *t*_(19)_ = 1.48, *p* = 0.16, when the TBF stimulus was old. Full results are available in Figure 4B.

For GLM2, results revealed a significant main effect of time, *F*_(1, 19)_ = 161.38, *p* < 0.001, $\eta_{\text{p}}^{2}$ = 0.90, suggesting greater Right PPA activation before the cue compared to after the cue. There was also a significant main effect of cued stimulus, *F*_(1, 19)_ =243.59, *p* < 0.001, $\eta_{\text{p}}^{2}$ = 0.56, suggesting greater activation when a building was cued compared to when a face was cued. There were no other significant effects (*p*s > 0.27).

Overall, these results are similar to the results from the left PPA and suggest that participants deprioritized buildings when faces were cued. Furthermore, increased memory stability (old TBF stimulus) reduces, but does not eliminate, this prioritization. The notable finding regarding the Right PPA compared to the left depended on whether the TBF stimulus was old or new. In the left PPA, participants were less likely to effectively utilize the cue to prioritize cued information when the TBF stimulus was old.

#### 3.2.3 FFA activation

GLM1 results revealed no main effect of the cued stimulus, *F*_(1, 19)_ = 1.51, *p* = 0.24, $\eta_{\text{p}}^{2}$ = 0.07 or TBF status, *F*_(1, 19)_ = 0.02, *p* = 0.89, $\eta_{\text{p}}^{2}$ = 0.001, but there was a significant interaction between the cued stimulus and TBF status, *F*_(1, 19)_ = 10.01, *p* = 0.005, $\eta_{\text{p}}^{2}$ = 0.35. We conducted paired samples *t*-tests to examine the significant interaction. The interaction was driven by greater FFA activation on trials when buildings were cued than trials when faces were cued, but only when the TBF stimulus was new, *t*_(19)_ = 2.86, *p* = 0.011. There were no differences in activation between the cued stimuli when the TBF stimulus was old, *t*_(19)_ = 0.50, *p* = 0.62.

**Figure 5:**
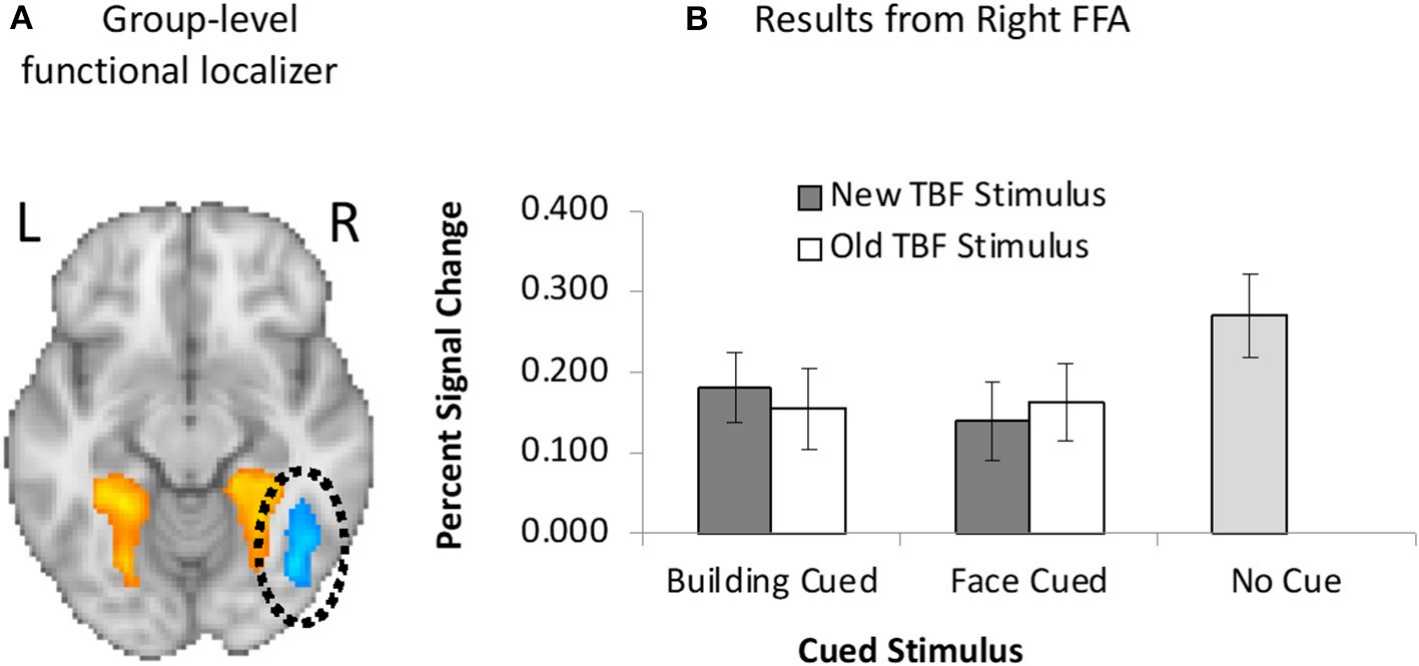
**(A)** Unilateral FFA (blue) activation resulting from the group-level functional localizer task. **(B)** Neuroimaging results from the left FFA when faces were cued or buildings were cued, and the TBF stimulus was new or old. Activation on no-cue trials is added for comparison. Error bars represent 95% confidence intervals.

We further examined FFA activation by comparing activation on no-cue trials to cue trials. When the TBF stimulus was new, there was significantly higher FFA activation on no-cue trials than trials when faces were cued, *t*_(19)_ = 6.50, *p* < 0.001, and when buildings were cued, *t*_(19)_ = 4.11, *p* = 0.001. The same pattern was observed when the TBF stimulus was old, in that no-cue trials resulted in higher FFA activation than trials when faces were cued, *t*_(19)_ = 4.55, *p* < 0.001, and when buildings were cued, *t*_(19)_ = 4.96, *p* = 0.001. Full results are available in Figure 5.

For GLM2, results revealed a significant main effect of time, *F*_(1, 19)_ = 70.77, *p* < 0.001, $\eta_{\text{p}}^{2}$ = 0.79, suggesting greater FFA activation before the cue compared to after the cue. There were no other significant effects for GLM2 in the FFA (*p*s > 0.43). Overall, these results are inconsistent with the PPA results, and suggest that face stimuli may be more challenging to prioritize in a directed forgetting paradigm.

#### 3.2.4 Whole brain analysis of pre vs. post cue

**Figure 6:**
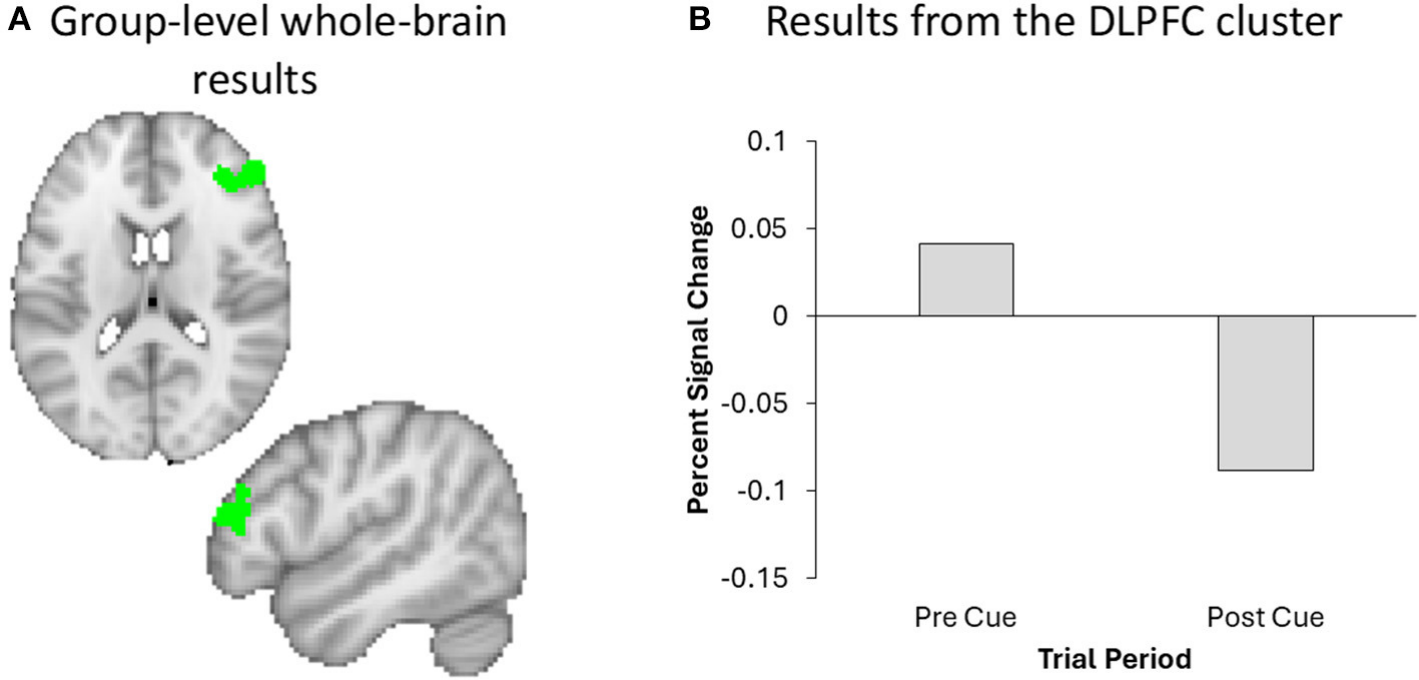
A Whole brain analysis revealed significant DLPFC activation (green) in the right hemisphere **(A)**, the effect of which plotted for visualization and inspection purposes only **(B)**.

We performed a whole-brain analysis in order to adjudicate between the distinct predictions for active and passive forgetting regarding VWM load using GLM2. Most notably, results revealed significant activation in the right DLPFC (Figure 6). The full cluster list for the whole-brain analysis of GLM2, pre-cue vs. post-cue, is presented in Table 1. There were no significant clusters of activity showing greater post-cue vs. pre-cue activity.

**Table 1:** Cluster # = the number of a cluster, ordered by size; Cluster k = the number of contiguous voxels in the cluster; Brain region = the region of local maxima included in the broader cluster.

**Cluster #**	**Cluster k**	**Brain region**	**H**	**Z**	**#**	**MNIx**	**MNIy**	**MNIz**
**Pre – Post Cue**								
1	68,670	Amygdala	L	4.26	174	−22	−8	−16
		Amygdala	R	4.36	192	18	−4	−18
		Central opercular cortex	L	4.82	480	−42	−2	12
		Cingulate gyrus anterior division	L	4.35	614	−4	16	32
		Cingulate gyrus anterior division	R	4.26	463	2	32	16
		Cingulate gyrus posterior division	L	4.03	369	−10	−48	4
		Cingulate gyrus posterior division	R	3.98	321	8	−48	6
		Hippocampus	L	4.73	374	−20	−40	4
		Hippocampus	R	4.96	457	20	−16	−16
		Insular cortex	L	4.90	265	−38	0	8
		Insular cortex	R	4.69	300	38	2	8
		Lateral occipital cortex inferior division	L	5.76	806	−38	−86	6
		Lateral occipital cortex inferior division	R	5.84	813	46	−84	−6
		Lateral occipital cortex superior division	L	5.33	1,417	−32	−86	26
		Lateral occipital cortex superior division	R	5.01	1,477	44	−74	22
		Lingual gyrus	L	5.64	603	−22	−50	−10
		Lingual gyrus	R	6.07	627	20	−42	−12
		Occipital fusiform gyrus	L	5.82	254	−22	−74	−10
		Occipital fusiform gyrus	R	5.66	268	34	−72	−14
		Occipital pole	L	5.51	795	−34	−94	0
		Occipital pole	R	5.12	608	20	−98	−6
		Postcentral gyrus	L	5.11	612	−20	−38	76
		Postcentral gyrus	R	4.37	384	66	−18	26
		Precuneous cortex	L	5.35	588	−12	−54	6
		Precuneous cortex	R	4.96	638	10	−58	12
		Temporal fusiform cortex posterior division	L	4.54	232	−32	−40	−24
		Temporal fusiform cortex posterior division	R	5.61	216	32	−34	−18
		Temporal occipital fusiform cortex	L	5.48	220	−40	−52	−18
		Temporal occipital fusiform cortex	R	6.40	381	32	−44	−18
		Temporal pole	R	3.74	281	44	14	−30
		Thalamus	L	4.18	150	−18	−32	−4
		Thalamus	R	4.57	150	16	−32	−2
2	385	Frontal Pole (DLPFC)	R	4.18	99	50	40	14
**Post – Pre**								
		No significant clusters						

The region names are taken from the Harvard Oxford atlas in FSL; H = principal hemisphere of the cluster, right (R) or left (L); Z = maximum z-value from the cluster within the given brain region; # = number of voxels from the cluster inside the given brain region. NB – for Cluster 1 above, we report cortical regions containing at least 300 voxels, plus subcortical regions with at least 150 voxels; MNI(X,Y,Z) = coordinates of the voxel with the maximum effect in the standardized space of the Montreal Neurological Institute (MNI), represented in units of millimeters (mm). Please note that images are resampled to the dimensions of the MNI 2 mm atlas (2 × 2 × 2 mm).

Next, we used the DLPFC cluster from GLM2 and performed an ROI analysis using the GLM1 results to evaluate whether DLPFC activity was differentially affected by the different trial-types. A 2 × 2 repeated measures ANOVA with cued stimulus (face cued, building cued) and TBF status (old, new) as the factors revealed no significant main effects or interaction for the DLPFC (*p*s > 0.16). Additionally, we compared DLPFC activation on cue vs. no-cue trials across the whole trial period (e.g., from the onset of the pre-change display to the offset of the final fixation cross), as was done for the PPA and FFA analyses. Results revealed no significant differences in DLPFC activation between cue and no cue trials, *t*_(19)_ = 0.62, *p* = 0.54.

**Figure 7:**
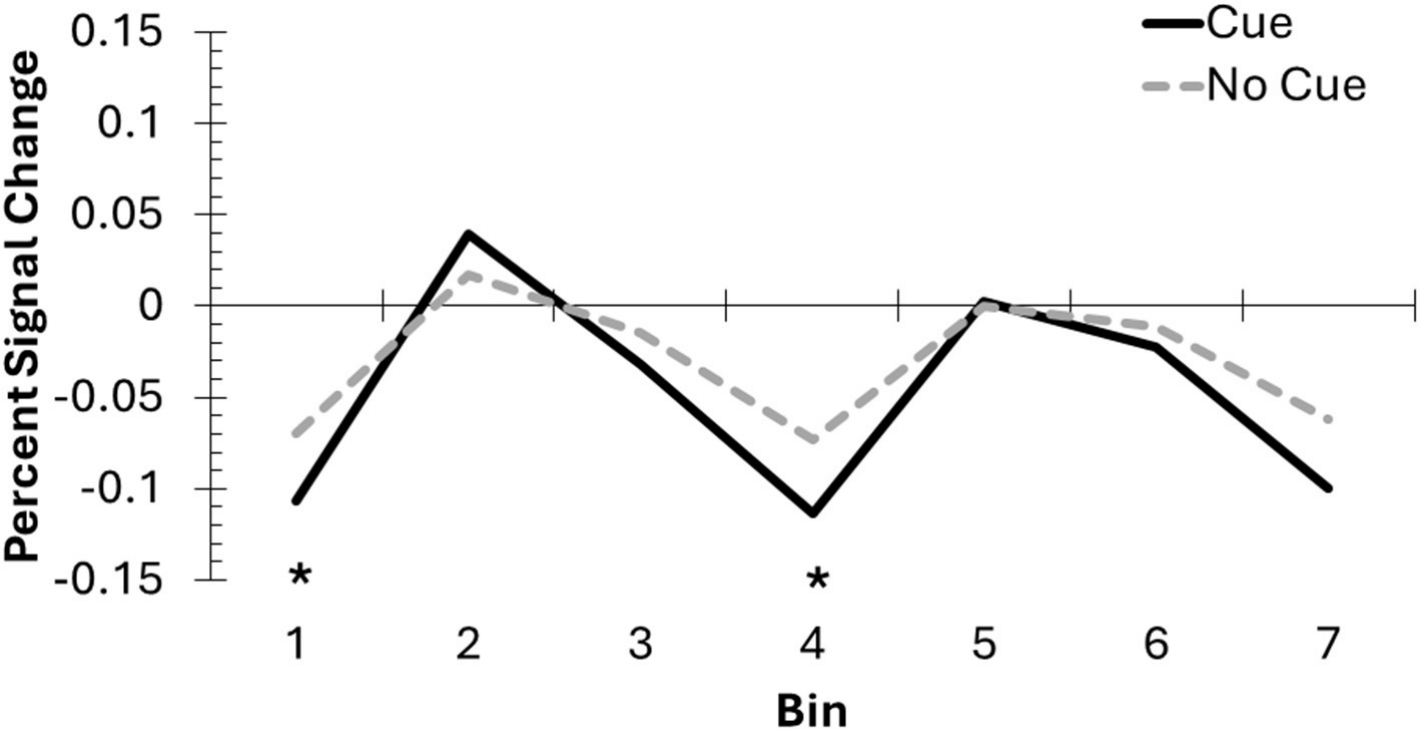
The DLPFC mask created from the whole brain analysis was used to conduct an exploratory FIR analysis, involving 2-second bins across the DF task to compare cue and no-cue trials. Incorporating the hemodynamic delay, Bins 2–4 likely represent activation from the pre-change display into the post-cue delay, before the onset of the post-change display response screen. Asterisks indicate significant differences between cue and no-cue trials.

For the FIR analyses, we used the DLPFC cluster produced from GLM2 to confirm the time course of the BOLD response. There were significant main effects of bin, *F*_(6, 114)_ = 4.89, *p* < 0.001, $\eta_{\text{p}}^{2}$ = 0.21, and trial type, *F*_(1, 19)_ = 7.89, *p* = 0.01, $\eta_{\text{p}}^{2}$ = 0.29, as well as a significant interaction, *F*_(6, 114)_ = 2.21, *p* = 0.047, $\eta_{\text{p}}^{2}$ = 0.10. The significant interaction was driven lower DLPFC activation on cue trials, compared to no-cue trials, at Bin 1, *t*_(19)_ = 2.26, *p* = 0.04, and importantly, Bin 4, *t*_(19)_ = 2.48, *p* = 0.02 (see Figure 7). There were no significant differences between cue and no-cue trials for Bins 2, 3, 5, 6, or 7 (*p*s > 0.11). Comparisons across bins for each trial type are available in Table 2. Overall, these results suggest that DLPFC activation changed throughout the trial for cue and no-cue trials, and the overall pattern of results between the two trial types is similar.

**Table 2:** Paired sample *t*-test results from the FIR analysis in the DLPFC.

	**Cue trials**		**No cue trials**	
	*t*_(19)_	***p***	*t*_(19)_	***p***
Bin 1 vs. Bin 2	**3.85**	**0.001**	**2.49**	**0.02**
Bin 2 vs. Bin 3	1.87	0.08	0.94	0.36
Bin 3 vs. Bin 4	1.92	0.07	1.82	0.09
Bin 4 vs. Bin 5	**3.13**	**0.005**	**2.44**	**0.03**
Bin 5 vs. Bin 6	0.82	0.42	0.47	0.65
Bin 6 vs. Bin 7	**2.80**	**0.01**	1.44	0.17

Bold values indicate significant effects.

## 4 Discussion

The current study attempted to document a mechanism of directed forgetting in VWM and determine whether forgetting is impacted by existing memory traces in LTM. There is disagreement among researchers if the mechanism of DF in VWM is due to passive forgetting or active suppression. The current study examined the pattern of brain activation associated with a DF task and found changes in activation in the PPA and DLPFC throughout the trial period. In terms of existing memory traces in LTM, results from the PPA suggested that brain activation is higher for cued information than non-cued information, and the difference is larger for representations that do not rely on LTM (i.e., the TBF stimulus has not been seen before). Previous research suggests that familiar stimuli elicit greater activation than unfamiliar stimuli (Weibert and Andrews, 2015), however, this may depend on the delay between presentations and the type of task (Henson, 2016). In the current study, participants were able to more effectively prioritize cued information when the TBF information had no previous memory trace. These results are most likely due to unfamiliar information (new TBF stimuli) leading to less brain activation than familiar information (old TBF stimuli).

One explanation for the results of the current study is that forgetting occurs through a passive forgetting process, due to changes in DLPFC activation. The DLPFC has previously been associated with cognitive effort (i.e., trying to actively suppress information; Soutschek and Tobler, 2020) and working memory load (Barbey et al., 2013; Yoon et al., 2016; i.e., dropping a stimulus from VWM), among other functions. Our results revealed that DLPFC activation decreased following the cue (see Figure 6 and Table 1), which may suggest that participants were no longer actively maintaining the TBF information in VWM. However, it is important to acknowledge that the DLPFC is involved with several cognitive functions, not just VWM load. Thus, there are several alternative explanations for changes in DLPFC activation, which we discuss below.

We conducted an FIR analysis to better understand how DLPFC activation changed throughout the trial. We hypothesized that DLPFC activation would decrease after the cue was presented, for cue trials, as the VWM load is greater for no-cue trials (maintain two stimuli for the whole trial) than cue trials (maintain one stimulus after the cue). Indeed, we observed a significant interaction between trial type (cue, no-cue) and bin, suggesting that the time course of forgetting differed between the two trial types. However, these significant effects in the DLPFC are not sufficient for determining the mechanism of forgetting in VWM. There are several alternative explanations for this shift in DLPFC activation throughout the time course of the trial.

First, there is a drastic change in the perceptual information available throughout the trial sequence, with two detailed stimuli presented initially, and then only a fixation cross and/or an arrow cue presented (depending on the trial type), until the post-change display appears. The decrease in DLPFC activation that we observed may have been due to differences in perceptual information throughout the trial (Lamichhane and Dhamala, 2015). Second, there may have been a general decrease in activation throughout the trial as participant engagement and attention decreased, especially if motivation to perform well was low, due to the relatively simple task and small set size (Szatkowska et al., 2008). Third, it is possible that participants used active forgetting, but the process occurred at a fast enough rate that was not detected with the current neuroimaging methods. If active forgetting occurred very quickly, then we would expect a decrease in DLPFC activation after the initial, successful forgetting, as the VWM load decreased (Barbey et al., 2013; Yoon et al., 2016).

Overall, the results from the current study are not able to distinguish between active and passive forgetting. Additionally, it is important to acknowledge the limitations of the current study. For example, it is possible that the current study was underpowered, as the power analysis was conducted using behavioral data with different variables, rather than fMRI data. Indeed, the high variability in results throughout the study, and the lack of a behavioral directed forgetting effect in VWM (Figure 2), suggest that a larger sample may be needed. Additionally, the set size of the current study (two items) did not tax working memory capacity (Baddeley, 1992, 2003), thus, future research should increase the set size, to determine if these effects persist when VWM capacity is exceeded. Finally, future research should implement single-item trials to compare DLPFC activation of single item trials to post-cue activation on cue trials with two stimuli. This may provide further evidence as to whether information is being dropped from VWM completely.

### 4.1 FFA vs. PPA

Results from the PPA suggest that when all the information presented on a given trial was new, participants were able to prioritize the cued information, and deprioritize the non-cued information. However, the FFA results were less conclusive. Specifically, the FFA results suggest that participants were unable to deprioritize faces when buildings were cued. Additionally, no-cue trials consistently resulted in greater FFA activation than cue trials. These results suggest there may be a trade-off in the FFA between constant maintenance of a face (no-cue trials) and a shift to prioritizing the cued stimulus. Additionally, previous research suggests that the FFA is most sensitive to the perception of faces, and that attention may not impact FFA activation (Gazzaley et al., 2005). The FFA's insensitivity to shifts in attention may be why the FFA results differ from the PPA. Additionally, the FFA is also sensitive to task-switching (Wylie et al., 2004). For example, Wylie and colleagues found that when participants switched from a task with faces to a task without faces, they found equivalent FFA activation for both tasks. In the current study, the cue could be considered a task switch from maintaining both stimuli to maintaining one stimulus, thus causing the inconsistent pattern of results in the FFA. Furthermore, FFA activation is often considered purely perceptual. FFA activation during encoding can predict successful retrieval, but FFA activation during retrieval may not differ for hits and misses (Lehmann et al., 2004). The current study analyzed FFA activation across the entire encoding and maintenance period. Thus, it is possible that the FFA results are confounded by existing perceptual activation.

Additionally, participants may have used mental imagery to maintain mental representations of the presented face may have impacted FFA activation (Sunday et al., 2018). However, research in this area often distinguishes between anterior vs. posterior FFA. Weiner et al. (2010) found that the anterior and medial FFA regions are less susceptible to these effects of non-sensory processing, such as mental imagery. However, the current study did not differentiate between different subregions of the FFA, and thus, we are not able to rule out the possibility that mental imagery impacted the results on the current study. Future research should further explore directed forgetting in various FFA subregions.

Another potential explanation for the FFA results may be Load Theory (Lavie, 2005), which argues that there is more perceptual activity when under a high load. Lavie argued that under a high memory load, there are fewer resources left for the suppression of perceptual distractions. Thus, in the current study, it is possible that the load of maintaining detailed stimuli may have made it more challenging to disengage from the face stimuli, thus leading to greater FFA activation, even when the face was TBF. However, we acknowledge that the overall memory load in the current study was likely lower than VWM capacity (Baddeley, 1992, 2003).

Finally, it is possible that the inconsistencies between the FFA and PPA results are due to group defined ROIs. Although individually defined ROIs appear to increase the sensitivity (Nieto-Castañón and Fedorenko, 2012), we adopted a more conservative group defined ROI approach (Kryklywy et al., 2013) to increase the generalizability of the findings. Our approach also ensured that we did not need to exclude participants for failing to identify any of the ROIs (e.g., Said et al., 2011), which was especially important to us, given our limited sample size. However, it is possible that use of subject-specific ROIs in the FFA and PPA could increase the sensitivity in those regions, though this may come at the cost of generalizability. Nevertheless, this is an important limitation of the current study and future research should examine the differences in FFA and PPA activation during forgetting with both group and individually defined ROIs.

### 4.2 Directed forgetting vs. retro-cues

It is important to consider how the current study may be informed by literature outside of directed forgetting, such as the retro-cue effect (Griffin and Nobre, 2003). There are certainly similarities between the directed forgetting task used in the current study and retro-cue tasks. For example, the active forgetting account argues that directed forgetting is characterized by the actively prioritizing TBR stimuli (like the attention shift to cued stimulus in the retro-cue effect), but also the actively deprioritizing TBF stimuli, which is unique to directed forgetting literature (Fawcett and Taylor, 2008; Wylie et al., 2008; Nowicka et al., 2011; Rizio and Dennis, 2013). Alternatively, the passive forgetting described in the current study is identical to a retro-cue effect. In both instances, one stimulus is prioritized and mostly likely to be tested (100% valid in DF studies, <100% valid in retro-cue studies). Thus, one way to describe the passive forgetting in the current study is to describe it as a shift in attention from all stimuli (pre-cue delay) to a single stimulus (post-cue delay). We hypothesize that participants in the current study shifted attention to the TBR stimulus, and thus were passively forgetting the TBF stimulus. That is, the removal of attention and passive forgetting may be the same process. The lack of a behavioral cuing effect may be due to several factors, such as the small set size or the specific stimuli. Future research should consider if there is a way to distinguish between the retro-cue effect and passive forgetting, or whether they occur via the same mechanism.

### 4.3 Forgetting in VWM and LTM

DF is more commonly studied in LTM than VWM (MacLeod, 1975; Anderson and Green, 2001; Wylie et al., 2008; Nowicka et al., 2011; Rizio and Dennis, 2013; Zwissler et al., 2015; Fawcett et al., 2016), and LTM theories of directed forgetting were used to motivate the research questions in the current study. The research examining the mechanism of directed forgetting in LTM has found behavioral support for either active suppression (Wylie et al., 2008; Nowicka et al., 2011; Rizio and Dennis, 2013) or passive forgetting (Zwissler et al., 2015; Dagry and Barrouillet, 2017). However, most DF research using neuroimaging has found support for active suppression as the mechanism of directed forgetting in LTM (for exception see Experiment 4 in Zwissler et al., 2015).

We chose to rely on LTM theories of directed forgetting in the current study due to the lack of research examining forgetting in VWM. Researchers often segment human memory in VWM and LTM because these types of memory are associated with specific characteristics. VWM is a capacity-limited memory store (3–4 stimuli), that allows for visual information to be manipulated (Fukuda et al., 2010) and quickly accessed for a brief period of time (Atkinson and Shiffrin, 1968; Craik and Lockhart, 1972). Alternatively, LTM has a very large capacity and may be capacity unlimited (Standing, 1973; Brady et al., 2008). Additionally, information in LTM is not active (via continuous rehearsal) prior to the presentation of the appropriate retrieval cue (Atkinson and Shiffrin, 1968). Cognitive resources are more limited within VWM than LTM (Atkinson and Shiffrin, 1968), thus forgetting may occur differently in VWM compared to LTM.

The availability of resources is the critical difference between VWM and LTM when examining the mechanism of directed forgetting. Specifically, active suppression is a cognitively demanding task. Increased DLPFC activation is associated with increased cognitive effort and is the primary neurological indicator for suppression (Wylie et al., 2008; Nowicka et al., 2011; Rizio and Dennis, 2013). Cognitive resources may be more readily available in LTM, thus allowing for a more active forgetting process, such as suppression. In VWM, however, information must be actively rehearsed to remain active in VWM. When participants receive a cue to maintain only the TBR stimuli, it is advantageous to use a passive forgetting strategy, which is less cognitively demanding because VWM rehearsal is also cognitively demanding.

The current study may inform LTM DF research, specifically for more difficult, cognitively demanding LTM tasks. It is possible that individual differences among participants or various methodologies lead to more cognitively demanding tasks, thus leading some LTM researchers to find support for passive forgetting as the mechanism of directed forgetting. For example, individuals with smaller VWM capacities may be more likely to forget information differently than individuals with very large VWM capacities. Future research should continue to explore the mechanism of directed forgetting depending on the relationship between VWM and LTM.

## 5 Conclusion

The goal of the current study was to determine the mechanism of directed forgetting in VWM, and how forgetting may be impacted by existing LTMs. We found changes in PPA and DLPFC activation, and there were differences between cue and no-cue trials. However, we were unable to definitively conclude the mechanism of directed forgetting and have instead offered several potential explanations for our results. Future research should continue to explore the mechanisms of forgetting in VWM and LTM, and how the strength of memory representations impacts forgetting.

## Data availability statement

The raw data supporting the conclusions of this article will be made available by the authors, without undue reservation.

## Ethics statement

The studies involving humans were approved by the Louisiana State University Institutional Review Board. The studies were conducted in accordance with the local legislation and institutional requirements. The participants provided their written informed consent to participate in this study.

## Author contributions

KM: Conceptualization, Data curation, Formal analysis, Investigation, Methodology, Project administration, Resources, Software, Validation, Visualization, Writing – original draft, Writing – review & editing. MB: Conceptualization, Methodology, Project administration, Resources, Supervision, Writing – review & editing. SH: Formal analysis, Validation, Visualization, Writing – review & editing. SG: Conceptualization, Formal analysis, Funding acquisition, Methodology, Resources, Software, Supervision, Visualization, Writing – original draft, Writing – review & editing.

## Footnotes

^1^This memory took place for 15 of the 20 participants. Five participants did not complete the memory test due to time constraints.
